# Clinico-pathological relationship between androgen receptor and tumour infiltrating lymphocytes in triple negative breast cancer

**DOI:** 10.3332/ecancer.2021.1317

**Published:** 2021-11-11

**Authors:** Hagar Elghazawy, Joaira Bakkach, Thanaa Helal, Ahmed M Aref, Mohamed Kelany, Lamiaa E Abdallah, Fatma S Abdelbakey, Dalia Ali, Doaa Z Ali, Mai O Ahmed, Amer Ali Abd El-Hafeez, Pradipta Ghosh, Mohamed O Alorabi

**Affiliations:** 1 Department of Clinical Oncology, Faculty of Medicine, Ain Shams University, Cairo, 11591, Egypt; 2 Biomedical Genomics & Oncogenetics Research Laboratory, Faculty of Sciences and Techniques of Tangier, Abdelmalek Essaadi University, Tangier, 90 000, Morocco; 3 Department of Pathology, Faculty of Medicine, Ain Shams University, Cairo, 11591, Egypt; 4 Faculty of Biotechnology, October University for Modern Sciences and Arts (MSA), Giza, 12451, Egypt; 5 Department of Clinical Oncology, Electricity Hospital, Cairo, 11775, Egypt; 6 Pharmacology and Experimental Oncology Unit, Cancer Biology Department, National Cancer Institute, Cairo University, Cairo, 11796, Egypt; 7 Department of Cellular and Molecular Medicine, School of Medicine, University of California, San Diego, La Jolla, CA 92093, USA; 8 Department of Medicine, University of California, San Diego, La Jolla, CA 92093, USA; 9 Rebecca and John Moore Comprehensive Cancer Center, University of California, San Diego, La Jolla, CA 92037, USA; 10 Veterans Affairs Medical Center, La Jolla, CA 92161, USA; § Hagar Elghazawy and Joaira Bakkach had contributed equally to the work; a https://orcid.org/0000-0001-6839-4147

**Keywords:** triple negative breast cancer, TNBC, androgen receptor, AR, tumour infiltrating lymphocytes, TILs

## Abstract

**Background:**

Triple negative breast cancer (TNBC) is an aggressive subtype of breast cancer (BC) with ill-defined therapeutic targets. Androgen receptor (AR) and tumour-infiltrating lymphocytes (TILs) had a prognostic and predictive value in TNBC. The relationship between AR, TILs and clinical behaviour is still not fully understood.

**Methods:**

Thirty-six TNBC patients were evaluated for AR (positive if ≥1% expression), CD3, CD4, CD8 and CD20 by immunohistochemistry. Stromal TILs were quantified following TILs Working Group recommendations. Lymphocyte-predominant breast cancer (LPBC) was defined as stromal TILs ≥ 50%, whereas lymphocyte-deficient breast cancer (LDBC) was defined as <50%.

**Results:**

The mean age was 52.5 years and 27.8% were ≥60 years. Seven patients (21.2%) were AR+. All AR+ cases were postmenopausal (≥50 years old). LPBC was 32.2% of the whole cohort. Median TILs were 37.5% and 10% (*p* = 0.1) and median CD20 was 20% and 7.5% (*p* = 0.008) in AR− and AR+, respectively. Mean CD3 was 80.7% and 93.3% (*p* = 0.007) and CD8 was 75% and 80.8% (*p*= 0.41) in AR− and AR+, respectively. All patients who were ≥60 years old expressed CD20. LDBC was found to be significantly higher in N+ versus N− patients (*p* = 0.03) with median TILs of 20% versus 50% in N+ versus N−, respectively (*p* = 0.03). LDBC was associated with higher risk of lymph node (LN) involvement (odds ratio = 6; 95% CI = 1.05–34.21; *p* = 0.04).

**Conclusions:**

AR expression was evident in older age (≥50 years). Median CD20 was higher in AR− TNBC, while mean CD3 was higher in AR+ tumours. LDBC was associated with higher risk of LN involvement. Larger studies are needed to focus on the clinical impact of the relation between AR and TILs in TNBC.

## Background

Triple negative breast cancer (*TNBC*) is a challenging *heterogeneous* disease with distinct molecular *subtypes* that does not have receptors for oestrogen, progesterone hormones and the human epidermal growth factor receptor 2 (HER2) protein. *TNBC* was grouped into six molecular subtypes: basal-like (BL) 1, BL2, mesenchymal (M), mesenchymal stem-like (MSL), immunomodulatory (IM) and luminal androgen receptor (LAR) [1]. But thereafter, Lehmann *et al* [2] found that transcripts in the previously defined IM and MSL subtypes came from tumour-infiltrating lymphocytes (TILs) and tumour-associated stromal cells, respectively, and they reduced the number of TNBC molecular subtypes to four (BL1, BL2, M and LAR).

TILs play an essential role in predicting response to chemotherapy and improving clinical outcomes in breast cancer (BC). Moreover, as the immunotherapy landscape continues to evolve, there is interest in whether the immune system could be playing a more substantial role in TNBC specifically. The association between TNBC subtypes and the impact of TILs is still not fully understood. However, accumulating evidence from several studies indicates that intra-tumoural levels of TILs in TNBC are: a) predictive for response to neo-adjuvant chemotherapy and b) prognostic in patients treated with adjuvant chemotherapy, being correlated with improved overall survival (OS) and disease free survival (DFS) [3].

Besides the immune cell markers, the androgen receptor (AR), which controls the transcription of different genes including the immune response genes, has been recognised as a valuable biomarker in TNBC [4]. The AR expression was correlated with better survival outcomes in TNBC [5], albeit its clinical utility and immunological impact remain unclear. However, many opened questions still need to be answered such as what is the prevalence of AR positivity in TNBC and whether AR expression correlates with the mean TILs or with CD3, CD4, CD8, CD20 expression. Also, it remains not fully clear whether there is any relationship between the predominance of TILs and the age or stage. Here, we addressed these questions and explored the correlation between the AR expression and the total and differential TILs in TNBC.

## Methods

In this cross-sectional, pilot study, patients’ records were reviewed retrospectively to select patients with TNBC. TNBC was deﬁned based on the American Society of Clinical Oncology/College of American Pathologists (ASCO/CAP) recommendations (2010) [6], as tumours with negative (<1% of nuclear staining) oestrogen receptor (ER), progesterone receptor (PR) and lack HER2 receptor overexpression or oncogene amplification. From a cohort of 800 BC patients who were diagnosed in 2012, at the clinical oncology department, Ain Shams University; 10% (80 patients) were diagnosed as TNBC in this year; of whom 36 patients had available tumour paraffin tissue and medical records. The clinico-pathological data and survival outcomes were collected. Tumour (T), nodes (N) and metastases (M) (TNM) staging was done according to the seventh edition of the American Joint Committee on Cancer (AJCC). The study protocol was approved by the Research Ethics committee, Faculty of Medicine, Ain Shams University, Cairo, Egypt.

Pathological evaluation was performed by a dedicated pathologist (TH), who was blinded for the clinical data. Haematoxylin and eosin-stained sections were revised for the negativity of ER, PR and Her2 and assessed for the histologic type and grade of the tumour. Then, the sections were examined to quantify the stromal TILs according to the 2014 TILs International Working Group [7], where it was defined as the percentage of lymphocytes in direct contact with tumour cells. Lymphocyte-predominant breast cancer (LPBC) was defined as TILs ≥ 50%, while lymphocytic deficient breast cancer (LDBC) was defined as TILs < 50%.

Formalin-fixed, paraffin-embedded tissue specimens were available for the evaluation of both of AR and TILs in 28 patients, AR alone in 5 patients and TILs alone in 3 patients. The AR expression (Code 200M-18) was evaluated by immunohistochemistry (IHC), and considered positive if ≥1% nuclear staining of the tumour cells [4]. Also, immunostaining was performed for T cell markers CD3 (Code 00000 51564), CD4 (Code 104R-28), CD8 (Code 108M-98) and B cell marker CD20 (Code 00000 27500). All antibodies were ready to use, from Cell Marque, California, USA. CD3, CD4, CD8 and CD20 immunostaining results were evaluated as mean per centage of the stained lymphocytes in relation to the total lymphocytes in the whole tissue section. Then the mean (for CD3 and CD8) and median (for CD4 and CD20) were calculated. The primary aim of our study was to describe the expression of AR and immune cells (CD3, CD4, CD8 and CD20) in TNBC, and the percentage of TILs as well. The secondary aim was to correlate the clinico-pathological parameters with these biomarkers.

### Statistical analysis

Recorded data were analysed using the Statistical Package for Social Sciences, version 20.0 (SPSS Inc., Chicago, Illinois, USA). Quantitative data were expressed as mean ± standard deviation (SD) or median and interquartile range (IQR). Qualitative data were expressed as frequency and percentage. Independent-samples *t*-test of significance and Mann–Whitney (*z*) test were used to compare two means and non-parametric data, respectively. Analysis of Variance (ANOVA) test and Kruskal–Wallis test were used to compare more than two means and multiple-group comparisons in non-parametric data, respectively. Chi-square (*x*^2^) test was used in order to compare proportions between qualitative parameters. As multivariate analysis is not suitable for small sets of data, estimates are represented according to univariate analysis. Spearman’s correlation coefficient (*r*) test was used to assess the degree of association between two sets of variables. The *p*-value was considered significant if ≤0.05.

## Results

### Patient characteristics

Thirty-six TNBC patients with available enough tumour material were identified for analysis. The patients’ characteristics are shown in Supplementary Material, Table S1. The mean age at diagnosis was 52.5 years (range: 30–75 years), and 27.8% of cases were ≥60 years old. Most of the tumours (58.﻿3%) were of invasive duct carcinoma (IDC) type, while medullary carcinoma and invasive lobular carcinoma (ILC) accounted for 22% and 11%, respectively. Grade II and III tumours were 30.6% and 52.8%, respectively. Stages I, II, III and IV represented 5.6%, 30.5%, 52.7% and 8.3%, respectively. Lymph nodes (LNs) were positive in 77.8% (28 patients). After a median follow-up of 39 months, nine patients had developed a disease progression and the 3-year OS was reached in 44.4% of the patients.

### AR expression and its relation with the clinico-pathological and survival parameters

AR was tested in 33 patients and it was expressed in 21.2% (7 patients). All AR+ cases (100%) were postmenopausal (≥50 years old). Although patients with AR+ tumours were older than those who were AR− (mean age: 55 versus 51.6 years), there was no statistically significant difference in age between the two groups (*p* = 0.47). LNs were involved in 77% and 85.7% in AR− and AR+, respectively, (*p* = 0.61). No statistical difference was found in median OS between AR− and AR+ groups (31.5 versus 25 months, *p* = 0.77). The clinico-pathological parameters according to AR expression are shown in Table 1.

**Figure 1.**
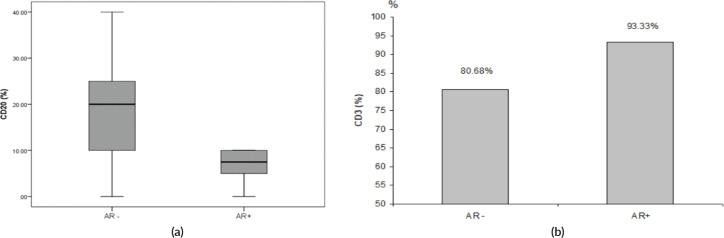
(a): Relation between AR and median CD20 expression (*p* = 0.008). (b): Relation between AR and mean CD3 expression (*p* = 0.007).

The majority of AR+ tumours (85.7%) was LDBC subtype, with median percentage of TILs was 37.5% and 10% in AR− and AR+ tumours, respectively, (*p* = 0.10). Median CD20 was significantly higher in AR− versus AR+ (20% versus 7.5%, respectively, *p* = 0.008), as depicted in Figure 1a, while mean CD3 was significantly lower in AR− versus AR+ (80.7% versus 93.3%, respectively, *p* = 0.007), as depicted in Figure 1b. On the other side, median CD4 and mean CD8 were not statistically different between AR− and AR+ tumours. Table 2 illustrated the correlation between the AR and total & differential TILs expression.

### Total and differential TILs expression and its relation with the clinico-pathological parameters

**Figure 2.**
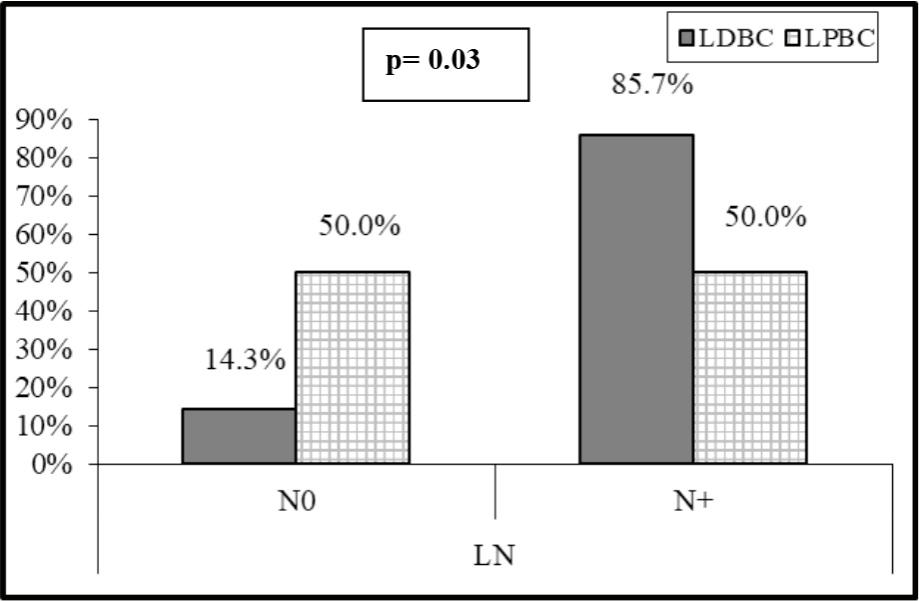
Relation between LN involvement and lymphocytic predominance.

In the 31 patients, where TILs were evaluated, the median TILs were 30% (range = 1%–70%), while LDBC and LPBC were 67.7% and 32.3%, respectively. CD20 and CD4 were negative in 9.6% and 54.8%, respectively. Table 3 showed descriptive analysis of the total and differential TILs expression. When correlating the lymphocytic predominance with the clinico-pathological parameters (shown in Table 4), LDBC type was found to be significantly higher in N+ versus N− patients (*p* = 0.03), as depicted in Figure 2. Median TILs were 20% versus 50% in N+ versus N−, respectively, (*p* = 0.03) as illustrated in Table 5a and b. Total TILs expression < 50% (LDBC) was associated with higher risk of LN involvement (odds ratio (OR) = 6; 95% CI = 1.05–34.21; *p* = 0.04).

**Figure 3.**
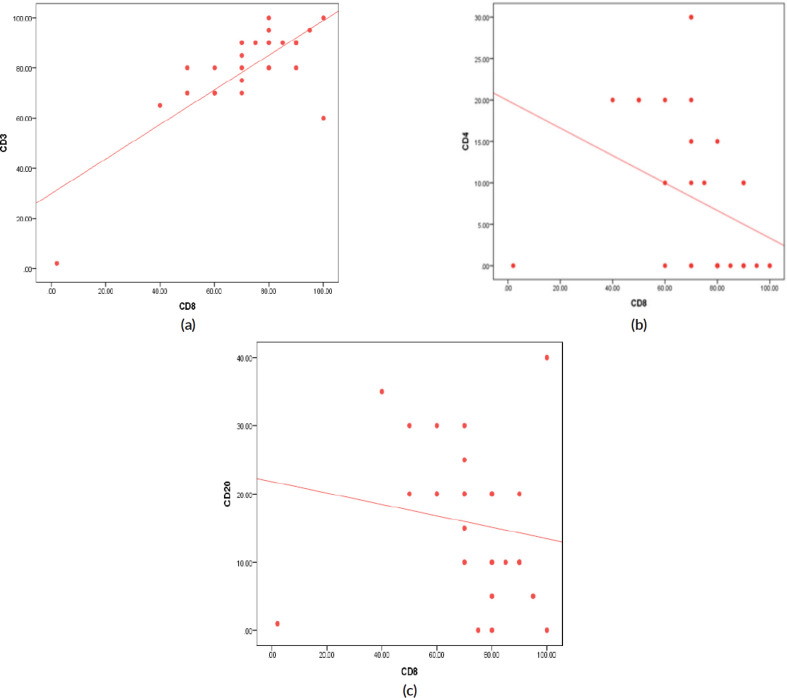
(a): Correlation between CD3 and CD8 expression (positive) (*r* = 0.591, *p* ≤ 0.001). (b): Correlation between CD4 and CD8 expression (inverse) (*r* = −0.527, *p* = 0.002). (c): Correlation between CD20 and CD8 expression (inverse) (*r* = −0.382, *p* = 0.034).

On the other side, when analysing the relationship between the age and TILs (Supplementary Material, Table S2), it was found that median TILs were lower in patients ≥60 years old despite statistically not significant, (median TILs = 10% versus 38% in ≥60 years old versus <60 years old, respectively, *p* = 0.45). Moreover, all patients who were ≥60 years old expressed B-cell marker (100%) (shown in Supplementary Material, Table S3). Furthermore, a significant positive correlation was present between CD8 and CD3 (correlation coefficient (*r*) = 0.591, *p* ˂ 0.001), while significant inverse correlations were present between CD3 and CD20 (*r* = −0.814, *p* ˂ 0.001), CD8 and CD20 (*r* = −0.382, *p* = 0.03) and CD8 and CD4 (*r* = −0.52, *p* = 0.002), as illustrated in Figure 3a–c and Supplementary Material, Table S4.

## Discussion

It is well established that the expression of AR differs according to molecular subtypes of BC with more frequent expression in ER negative cancers. The prevalence of AR+ expressing tumours is generally ranging from 10% to 41% in TNBC cases [1, 8–14], with rare reports showing rates up to 79% [15, 16]. In accordance with most published reports, our rate of AR expression in TNBC was 21.2%.

Whether clinico-pathologic characteristics of TNBC vary based on AR expression status have been extensively studied [8, 13, 15, 17, 18]. Some studies showed that patients with AR+ tumours were significantly older, exhibited tumours with significantly lower grades (I–II), more frequent nodal involve ment, non-ductal histology and lower Ki67 [14, 15, 17, 18]. Other reports described reduced LN metastases in AR+ TNBCs [8], or just similar clinico-pathologic profile between AR+ and AR− TNBC tumours [13]. Herein, there was no statistically significant difference in the clinico-pathological parameters according to AR expression. However, AR+ cases were older in age and exhibited more regional nodal spread. Despite statistically insignificant, this profile was analogous to the LAR subtype described by Lehmann *et al* [2].

Available evidence about the prognostic value of AR in TNBC is controversial. Some reports suggested that AR-positivity was associated with good outcomes [8, 13], whereas others concluded that AR status conferred worse prognosis [19] or had no significant impact on disease prognosis [4, 20, 21]. Many factors may explain these discrepant results across studies including the sample size limited cohorts, differences in the ethnic origin, the anti-AR antibodies used for staining, staining/scoring method, as well as variability in the thresholds used to define AR positivity [4]. A meta-analysis published in 2017, demonstrated that AR-positive status was associated with better DFS and OS in TNBC (hazard ratio (HR) = 0.64; 95% CI = 0.51–0.81; *p* < 0.001 and HR = 0.64; 95% CI = 0.49–0.88; *p* < 0.001, respectively), in univariate analysis [5]. Of note, no multivariate analysis was provided and this meta-analysis included heterogeneous studies in terms of methods of AR scoring, clinical cohorts’ characteristics, therapies received and length of follow-up. A large multi-institutional study including about 1,407 TNBC tumours issued after this meta-analysis concluded that the AR-positivity was a marker of good prognosis in USA and Nigerian cohorts, whereas it conferred poor prognosis in Norway, Ireland and Indian cohorts, and was neutral in UK cohort [4]. Whereas a more recent meta-analysis (2020) [21] demonstrated that AR expression in TNBC was not associated with DFS (HR = 0.92; 95% CI = 0.67–1.27; *p* = 0.63), OS (HR = 0.91; 95% CI = 0.67–1.22; *p* = 0.53), distant-DFS (HR = 1.02; 95% CI = 0.96–1.08; *p* = 0.48) or recurrence-free survival (HR = 0.95; 95% CI = 0.46–1.98; *p* = 0.90), regardless of the confounding factors and heterogeneity that existed among included studies. Our study results had matched the latter meta-analysis results, where no statistical difference in median OS (31.5 versus 25 months, *p* = 0.77) or relapse/progression rate (26.9% versus 14.3%, *p* = 0.48) was found between AR− and AR+ groups.

Importantly﻿, the presence of more frequent special histological subtypes with poor prognosis as medullary carcinoma, ILC and adenoid cystic carcinoma in the AR− versus the AR+ group (42% versus 28%) in our cohort, may have an impact on survival as pointed to by other studies [22].

Compared to other subtypes, TNBC was shown to exhibit higher levels of TILs [23]. There is heterogeneity of TILs cut-off used in published studies in order to distinguish between LPBC and LDBC. Some studies defined LPBC as showing more than 50% of lymphocyte infiltration [24, 25], whereas others used different cut-offs [26]. In our cohort, median TILs were 30% (range: 1%–70%), with a LPBC prevalence of 32.2%, which is not in full agreement with other reports. Adams *et al* [24] reported much lower median TILs percentage (10%), and with using the same cut-off of ≥50% TILs, only 4.4% were LPBC, whereas Pruneri *et al* [25] described a median TILs level of 20%, with LPBC prevalence of 22% of cases.

Little is known about the association between TNBC clinico-pathologic features and lymphocytic predominance. A pooled analysis of nine large studies by Loi *et al* [26] demonstrated that TILs were significantly lower in older age. Whilst, Adams *et al* [24] reported no strong associations between TILs scores and age or menopausal status. Despite not statistically significant, we showed lower median TILs in patients ≥60 years versus <60 years old (10% versus 38%, *p* = 0.45).

Interestingly, we found that patients with LN involvement were significantly more likely to be LDBC, where a total TILs expression < 50% (LDBC) was associated with higher risk of LN involvement (OR = 6; 95% CI = 1.05–34.21; *p* = 0.04). This is in agreement with Loi *et al* [26], but in contrast to a recent meta-analysis which concluded that no significant association between decreased TILs and LN metastasis risk [27].

Our knowledge about the association between TILs and AR is still limited. In a large cohort study about non-metastatic TNBC of LAR subtype, this tumour subset was found to exhibit lower median stromal TILs and to be less likely LPBC (≥50% TILs) compared to non-LAR, although this did not reach statistical significance [28], similarly to our study. However, we did not examine the genetic profiles of our AR+ tumours to classify them into the LAR subtype. Other reports using IHC described significant association between AR expression and lower levels of stromal TILs [11, 17].

Studies about the immune cells subsets composition of TILs according to AR expression are very scarce. In our study, median CD20 was significantly higher in AR− tumours compared to those with AR+ (20% versus 7.5%, respectively, *p* = 0.008). Whereas, mean CD3 was significantly lower in AR− versus AR+ (80.7% versus 93.3%, respectively, *p* = 0.007). On the other side, previous publications reported that CD8+ were more frequent in AR+ than AR− tumours [12, 29, 30], in contrast to our study which showed that neither CD8 nor CD4 were statistically different between AR+ and AR− tumours.

Based on two large-scale BC genomics data, evidence from a comprehensive analysis of 26 immune gene-sets including 15 immune cell type and function suggested that TNBC had the strongest tumour immunogenicity. Comparison of the immune infiltrate densities of different immune cell subpopulations demonstrated higher degree of infiltration in TNBC than non-TNBC, including CD3, CD8 and CD20 and others [31].

T-lymphocytes represent the main lymphocyte type in the tumour microenvironment, and the majority of T lymphocytes express a cytotoxic effector phenotype (CD8+). Intra-tumoural and adjacent stromal CD8+ T-cell infiltration have been found to be significantly associated with ER negativity and basal phenotype [32, 33]. Infiltrating CD8+ T-cells have been reported in more than 60% of TNBC cases [33, 34]. In our study, CD8 was expressed in 100% of the cases with the mean of its expression was 73.4%.

The role of tumour-infiltrating B cells (CD20) as components of TILs in BC subtypes is still unclear. A positive correlation between higher numbers of total CD20+ B cells and ER and PR negativity, and basal phenotype has been reported [35]. In our study, CD20 was expressed in 90.3% of the tumours and its median expression was significantly higher in AR− versus AR+ TNBC (20% versus 7.5%, respectively, *p* = 0.008).

Using a digital pathology computational workflow to quantify the spatial patterns of five immune markers (CD3, CD4, CD8, CD20 and FoxP3) in TNBC, Mi *et al* [36] demonstrated positive correlations between CD3 and CD8 cells. Similarly, we also showed a significant positive correlation between CD3 and CD8. Data from a study that used multiplexed ion beam imaging to simultaneously quantify *in situ* expression of 36 proteins in 41 patients with TNBC, suggested that all patients with B cells had also CD4 T cells and CD8 T cells [37]. In contrast, we found in our study significant inverse correlations between CD20 and CD8, as well as CD20 and CD3.

Immune cellular subpopulations in BC representing the innate immunity (natural killer, CD68+ and CD11c+ cells) and adaptive immunity (CD3+ cells (CD8+ or CD4+) and CD20+ cells) [38], worth thorough evaluation in TNBC, with the aim of understanding its clinical implications in BC management. In a recent consensus report for the management of TNBC, the majority of the panellists concluded that more evidence to support the predictive value of TILs and its impact on the clinical decision are warranted [39].

As no prior studies evaluating the exact relation between AR and TILs in this unique disease entity ‘TNBC’, we tended to represent all the statistical analyses and correlations which we evaluated, keeping with the aim of this exploratory study which may help future research. This study had mainly described the expression patterns of AR and TILs in TNBC. Moreover, the correlation between AR and the total and different TILs subpopulations was illustrated. TILs were evaluated by one pathologist who was blinded to the clinical characteristics and according to the International Working Group. However, our findings should be interpreted carefully. The limitations of our study include: i) the retrospective nature, ii) the small sample size (despite this, there were some significant correlations) and iii) the survival data was not mature due to the short follow-up duration (median: 39 months).

## Conclusions

This study highlighted the probable relationship between the AR and total and differential TILs expression in TNBC; and the clinico-pathological characteristics as well. Understanding the immune micro-environment in a subset of tumours with poor prognosis and less identified therapeutic targets like TNBC, may pave the way for the advent of immunotherapy in specific group of patients. Moreover, lower TILs density may identify a subpopulation of TNBC who warrants more radical regional LNs management. The prognostic relevance and the potential predictive impact of AR and TILs in TNBCs merit further evaluation in larger scale studies.

## Conflicts of interest

All authors declared no conflicts of interest.

## Authors’ contributions

Conception and design: HE, JB, TH, AMA, MK, DZA, MO Ahmed, MO Alorabi

Acquisition and interpretation of data: all authors

Drafting the manuscript and revising it critically for important intellectual content: all authors.

## Funding

None.

## Figures and Tables

**Table S1.** Patients’ characteristics of the whole cohort (36 patients).

Patients’ characteristics		No = 36	%
Age category	Age < 60 years Age ≥ 60 years	26 10	72.2 27.8
Menopausal status	Pre-menopausal	11	30.5
	Post-menopausal	25	69.5
Laterality	Bilateral	2	5.6
	Right	17	47.2
	Left	16	44.4
	Unknown	1	2.8
Pathology	IDC Medullary carcinoma ILC Adenoid cystic Unknown	21 8 5 1 1	58.3 22.2 13.9 2.8 2.8
Grade	Grade II	11	30.6
	Grade III	19	52.8
	Unknown	6	16.6
Neo-adjuvant chemotherapy	Negative	31	86.1
	Positive	3	8.3
	Unknown	2	5.6
Type of surgery	MRM	30	83.3
	BCS	6	16.7
Adjuvant chemotherapy	Negative	3	8.3
	Positive	30	83.4
	Unknown	3	8.3
Adjuvant radiotherapy	Negative	7	19.4
	Positive	23	63.9
	Unknown	6	16.7
TNM staging	I II III IV Unknown	2 11 19 3 1	5.6 30.5 52.8 8.3 2.8
T stage	T1 T2 T3 T4 Unknown	5 18 7 5 1	13.9 50.0 19.4 13.9 2.8
N stage	N0 N1 N2 N3 Unknown	8 9 13 5 1	22.2 25.0 36.1 13.9 2.8
LN category	N0 N+	8 28	22.2 77.8
Relapse/progression	Negative	27	75.0
	Positive	9	25.0
	Unknown	0	0.0
Type of relapse (=9)	Systemic	6	66.7
	Regional	1	11.1
	Unknown	2	22.2
Systemic relapse (=6)	Liver Brain Lung, bone Unknown	1 1 2 2	16.6 16.6 33.4 33.4
3-year OS			
	<3 years ≥3 years	20 16	55.6 44.4

IDC, Invasive ductal carcinoma; ILC, Invasive lobular carcinoma; MRM, Modified radical mastectomy; BCS, Breast conservative surgery; LN, Lymph node; OS, Overall survival

**Table S2.** Relation between age and the total and differential TILs.

Total and differential TILs		Age		Test value	*p*-value
		Age < 60	Age ≥ 60		
Total TILs (%)	Median (IQR)	38 (10–50)	10 (3–50)	−0.745^b^	0.456
	Range	1–60	1–70		
Median CD20 (%)	Median (IQR)	18 (10–20)	10 (10–20)	−0.156^b^	0.876
	Range	0–40	1–35		
Mean CD3 (%)	Mean ± SD	83.64 ± 10.14	73.00 ± 28.54	1.554^a^	0.131
	Range	60–100	2–95		
Median CD4 (%)	Median (IQR)	5 (0–20)	0 (0–10)	−0.933^b^	0.351
	Range	0–30	0–20		
Mean CD8 (%)	Mean ± SD	76.36 ± 14.24	66.33 ± 29.00	1.302^a^	0.203
	Range	50–100	2–90		

IQR, Interquartile range; SD, Standard deviation

a Independent *t*-test

b Mann–Whitney test

**Table S3.** Relation between age and the total and differential TILs.

Total and differential TILs		Age				Chi-square test	*p*-value
		Age < 60		Age ≥ 60			
		No.	%	No.	%		
LDBC versus LPBC	LDBC	15	68.2	6	66.7	0.007	0.935
	LPBC	7	31.8	3	33.3		
CD20 expression	Negative	3	13.6	0	0.0	1.359	1.000
	Positive	19	86.4	9	100		
CD3 expression	Negative	0	0.0	0	0.0	NA	NA
	Positive	22	100.0	9	100		
CD4 expression	Negative	11	50.0	6	66.7	0.716	0.397
	Positive	11	50.0	3	33.3		
CD8 expression	Negative	0	0.0	0	0.0	NA	NA
	Positive	22	100.0	9	100		

N, Not applicable

**Table S4.** Correlation between the total and differential TILs expression. The p-value was considered significant if ≤0.05.

	Total TILs (%)		CD20 (%)		CD3 (%)		CD4 (%)		CD8 (%)	
	*r*	*p*-value	*r*	*p*-value	*r*	*p*-value	*r*	*p*-value	*r*	*p*-value
Total TILs (%)	-	-	0.228	0.218	-0.151	0.416	-0.008	0.964	-0.060	0.747
CD20 (%)	0.228	0.218	-	-	**−0.814**	**<0.001**	0.200	0.282	**−0.382**	**0.034**
CD3 (%)	**−**0.151	0.416	**−0.814**	**<0.001**	-	-	**−**0.101	0.590	**0.591**	**<0.001**
CD4 (%)	**−**0.008	0.964	0.200	0.282	**−**0.101	0.590	-	-	**−0.527**	**0.002**
CD8 (%)	**−**0.060	0.747	**−0.382**	**0.034**	**0.591**	**<0.001**	**−0.527**	**0.002**	-	-

**Table 1.** The clinico-pathological parameters according to AR expression.

Clinico-pathological parameters		AR− (26)		AR+ (7)		Chi-square test	*p*-value
		No.	%	No.	%		
Age	Mean ± SD Range	51.65 ± 11.76 30–75		55.00 ± 4.40 50–63		−0.732^a^	0.470
Age category	<60 years ≥60 years	18 8	69.2 30.8	6 1	85.7 14.3	0.755	0.385
Menopausal status	Pre-menopausal	9	34.6	0	0.0	3.332	0.068
	Post-menopausal	17	65.4	7	100		
Laterality	Right Left Bilateral Unknown	12 12 1 1	46.2 46.2 3.8 3.8	4 3 0 0	57.1 42.9 0.0 0.0	0.689	0.876
Pathology	IDC Medullary carcinoma ILC Adenoid cystic Unknown	14 7 3 1 1	53.9 26.9 11.6 3.8 3.8	5 1 1 0 0	71.4 14.3 14.3 0.0 0.0	3.476	0.627
Grade	Grade II	9	34.6	2	28.6	2.640	0.267
	Grade III	11	42.3	5	71.4		
	Unknown	6	23.1	0	0.0		
TNM staging	I II III IV Unknown	2 7 15 1 1	7.7 26.9 57.8 3.8 3.8	0 3 4 0 0	0.0 42.9 57.1 0.0 0.0	1.539	0.820
T stage	T1 T2 T3 T4 Unknown	5 10 6 4 1	19.2 38.5 23.1 15.4 3.8	0 5 1 1 0	0.0 71.4 14.3 14.3 0.0	3.139	0.535
LN category	N0 N+	6 20	23.1 76.9	1 6	14.3 85.7	0.255	0.614
Relapse/progression	Negative Positive Unknown	19 7 0	73.1 26.9 0.0	6 1 0	85.7 14.3 0.0	0.480	0.489
Median OS	Median (IQR) Range	31.5 (18–44) 1.5–216		25 (17–77) 4–86		−0.286^b^	0.775
3-year OS	<3 years ≥3 years	14 12	53.8 46.2	4 3	57.1 42.9	0.024	0.876

IDC, Invasive ductal carcinoma; ILC, Invasive lobular carcinoma; LN, Lymph node; IQR, Interquartile range; OS, Overall survival

a Independent *t*-test

b Mann–Whitney test

**Table 2.** The correlation between AR and total & differential TILs expression. The *p*-value was considered significant if ≤0.05.

Variables		AR− (26)	AR+ (7)	Test value	p-value
Median TILs (%)	Median (IQR)	37.5 (10–50)	10 (5–20)	−1.607^c^	0.108
	Range	1–70	3–40		
LPBC versus LDBC	LDBC	14 (53.8%)	6 (85.7%)	3.082^a^	0.214
	LPBC	8 (30.8%)	0 (0.0%)		
	Unknown	4 (15.4%)	1 (14.3%)		
Median CD20 (%)	Median (IQR)	20 (10–25)	7.5 (5–10)	−2.643^c^	**0.008**
	Range	0–40	0–10		
CD20 expression	Negative	2 (7.7%)	1 (14.3%)	0.290^a^	0.865
	Positive	20 (76.9%)	5 (71.4%)		
	Unknown	4 (15.4%)	1 (14.3%)		
Mean CD3 (%)	Mean ± SD	80.7 ± 10.1	93.3 ± 4.1	**−**2.954^b^	**0.007**
	Range	60–100	90–100		
CD3 expression	Negative	0 (0.0%)	0 (0.0%)	0.005^a^	0.943
	Positive	22 (84.6%)	6 (85.7%)		
	Unknown	4 (15.4%)	1 (14.3%)		
Median CD4 (%)	Median (IQR)	0 (0–10)	12.5 (0–20)	**−**1.435^c^	0.151
	Range	0–20	0–30		
CD4 expression	Negative	14 (53.8%)	2 (28.6%)	1.786^a^	0.409
	Positive	8 (30.8%)	4 (57.1%)		
	Unknown	4 (15.4%)	1 (14.3%)		
Mean CD8 (%)	Mean ± SD	75.0 ± 16.3	80.8 ± 10.2	−0.825^b^	0.417
	Range	40–100	70–95		
CD8 expression	Negative	0 (0.0%)	0 (0.0%)	0.005^a^	0.943
	Positive	22 (84.6%)	6 (85.7%)		
	Unknown	4 (15.4%)	1 (14.3%)		

IQR, Interquartile range; LPBC, Lymphocytic predominance breast cancer; LDBC, Lymphocytic deficient breast cancer

a Chi-square test

b Independent *t*-test

c Mann–Whitney test

**Table 3.** Descriptive analysis of the total and differential TILs expression.

Variables		No. = 31
Median TILs (%)	Median (IQR)	30 (10–50)
	Range	1–70
LDBC versus LPBC	LDBC	21 (67.7%)
	LPBC	10 (32.3%)
Median CD20 (%)	Median (IQR)	15 (10–20)
	Range	0–40
CD20 expression	Negative	3 (9.6%)
	Positive	28 (90.4%)
Mean CD3 (%)	Mean ± SD	80.5 ± 17.7
	Range	2–100
CD3 expression	Negative	0 (0.0%)
	Positive	31 (100%)
Median CD4 (%)	Median (IQR)	0 (0–15)
	Range	0–30
CD4 expression	Negative	17 (54.8%)
	Positive	14 (45.2%)
Mean CD8 (%)	Mean ± SD	73.4 ± 19.7
	Range	2–100
CD8 expression	Negative	0 (0.0%)
	Positive	31 (100%)

IQR, Interquartile range; SD, Standard deviation; LPBC, Lymphocytic predominance breast cancer; LDBC, Lymphocytic deficient breast cancer

**Table 4.** Relation between lymphocytic predominance and clinico-pathological parameters. The *p*-value was considered significant if ≤0.05.

Variables		LDBC (= 21)		LPBC (=10)		Chi-square test	*p*-value
Age	Age < 60	15	71.4%	7	70.0%	0.007	0.935
	Age ≥ 60	6	28.6%	3	30.0%		
Morphology	IDC Medullary ILC Adenoid cystic	12 5 3 1	57.1% 23.8% 14.3% 4.8%	6 3 1 0	60.0% 30.0% 10.0% 0.0%	0.873	0.928
TNM staging	I II III IV	2 6 11 2	9.5% 28.6% 52.4% 9.5%	0 5 4 1	0.0% 50.0% 40.0% 10.0%	2.045	0.563
N stage	N0	3	14.3%	5	50.0%	4.762	0.190
	N1	7	33.3%	2	20.0%		
	N2	9	42.9%	2	20.0%		
	N3	2	9.5%	1	10.0%		
LN category	N−	3	14.3%	5	50.0%	4.513	**0.034**
	N+	18	85.7%	5	50.0%		
OS	Median (IQR)	32	(18–72)	25	(12–39)	−0.993^a^	0.320
	Range	2–216		9–48			
3-year OS	<3-year OS	11	52.4%	6	60.0%	0.159	0.690
	≥3-year OS	10	47.6%	4	40.0%		

IDC, Invasive duct carcinoma; ILC, Invasive lobular carcinoma; LN, Lymph node; IQR, Interquartile range; OS, Overall survival

a Mann–Whitney test

**Table 5A.** Relation between LN involvement and the total and differential TILs. The *p*-value was considered significant if ≤0.05.

Total and differential TILs		LN involvement		Test value	*p*-value
		N− (= 8)	N+ (=23)		
Total TILs (%)	Median (IQR)	50 (38–55)	20 (5–40)	−2.159^b^	**0.031**
	Range	10–60	1–70		
Median CD20 (%)	Median (IQR)	15 (3–20)	15 (10–25)	−1.089^b^	0.276
	Range	0–20	0–40		
Mean CD3 (%)	Mean ± SD	85.6 ± 9.8	78.8 ± 19.6	0.940^a^	0.355
	Range	70–100	2–100		
Median CD4 (%)	Median (IQR)	5 (0–13)	0 (0–20)	−0.074^b^	0.941
	Range	0–20	0–30		
Mean CD8 (%)	Mean ± SD	74.4 ± 15.0	73.1 ± 21.4	0.151^a^	0.881
	Range	50–100	2–100		

LN, Lymph node; IQR, Interquartile range; SD, Standard deviation

a Independent *t*-test

b Mann–Whitney test

**Table 5B.** Relation between LN involvement and the total and differential TILs. The p-value was considered significant if ≤0.05.

Total and differential TILs		LN involvement				Chi-square test	*p*-value
		N−		N+			
		No.	%	No.	%		
LDBC versus LPBC	LDBC	3	37.5	18	78.3	4.513	**0.034**
	LPBC	5	62.5	5	21.7		
CD20 expression	Negative	2	25.0	1	4.3	2.896	0.089
	Positive	6	75.0	22	95.7		
CD3 expression	Negative	0	0.0	0	0.0	NA	NA
	Positive	8	100	23	100.0		
CD4 expression	Negative	4	50.0	13	56.5	0.102	0.750
	Positive	4	50.0	10	43.5		
CD8 expression	Negative	0	0.0	0	0.0	NA	NA
	Positive	8	100	23	100		

NA, Not applicable
